# No Effect of Selective Maturation on Fruit Traits for a Bird-Dispersed Species, *Sambucus racemosa*

**DOI:** 10.3390/plants10020376

**Published:** 2021-02-16

**Authors:** Kohei Koyama, Mayu Tashiro

**Affiliations:** Laboratory of Plant Ecology, Department of Agro-Environmental Science, Obihiro University of Agriculture and Veterinary Medicine, Inadacho, Obihiro 080-8555, Hokkaido, Japan

**Keywords:** fruit size, fruit-to-flower ratio, ornithochory, fruit dispersal, seed dispersal, animal dispersal, wider choice, frugivore, dispersal syndrome, fruit syndrome

## Abstract

Selective abortion, also called selective maturation, is a phenomenon wherein maternal plants selectively mature ovules that have the potential to grow into higher-quality fruits, such as those that contain more seeds. We hypothesized that the effects of selective maturation on fruit traits could be influenced by the dispersal mechanism. However, to date, limited studies have been conducted on selective maturation in bird-dispersed fruits. Unlike self- or wind-dispersed species, bird-dispersed species would not selectively mature fruits that contain more seeds because they are not preferred by birds. Here, we investigated the effect of selective abortion on the fruit traits of a bird-dispersed species, elderberry (*Sambucus racemosa* L. subsp. *kamtschatica*). We performed a flower-removal experiment. Half of the inflorescences on each individual tree were removed for the treatment group, whereas the control group was not manipulated. We found that the flower-removed trees showed higher fruit sets, suggesting the existence of resource limitation. The number of seeds per fruit did not increase by the experimental treatment. Additionally, the control individuals did not produce larger fruits. The lack of effects on fruit traits supported our hypothesis that the effect of selective maturation on fruit traits may differ among species with different dispersal mechanisms.

## 1. Introduction

Selective abortion is a phenomenon wherein maternal plants selectively abort lower-quality ovules or immature fruits before fruit maturation to mature only those fruits or seeds that are of a higher quality [1,2,3,4,5,6,7] and thereby save resources [3], though resource limitation is not the sole factor that controls abortion in plants [7,8,9,10,11,12,13,14,15]. The process of selective abortion also has been considered as a mode of developmental selection [5], which states that as a result of completion among genetically diverse embryos, higher-fitness ones, such as those with more vigor or more developmental stability [5,16], are selectively developed into mature seeds [8,17]. Two selective mechanisms can occur simultaneously: selective fruit abortion and selective embryo or seed abortion. In selective fruit abortion, ovaries or immature fruits that have a higher potential to mature into higher-quality fruit are selectively matured, while those with a lower potential are aborted [3,5,18,19,20,21,22,23,24]. In selective seed or embryo abortion, ovules within a single ovary that have the potential to mature into higher-quality seeds are selectively matured [25,26,27,28,29]. The existence of selective maturation has been confirmed via hand-thinning experiments (i.e., random removal of flowers, ovules, or immature fruits [18,30]), which are based on the wider-choice hypothesis. This hypothesis states that maternal plants that do not undergo hand-thinning have a wider choice of high-quality zygotes or embryos for selective maturation than plants that have experienced the artificial random removal of flowers [18,31,32]. Therefore, the wider-choice hypothesis predicts that fruits that escape self-thinning (i.e., spontaneous thinning by maternal plants [18,30]) are of higher quality than those escaped artificial random thinning [18].

Compared with numerous previous studies of the effects of selective abortion on seed and offspring traits, the effect of selective maturation on fruit traits has not been thoroughly investigated, and the results that have been published show species-dependent differences. We suggest that these differences could be explained in part by species-dependent dispersal mechanisms. For wind- or self-dispersed (i.e., ballistic explosion or gravitational fall) species, fruits that contain more seeds have been considered to be higher quality [3,7,18,23,33], because these fruits may minimize the cost of fruit production per seed [3,23] and also because the seeds from these are selected under more intense pollen tube competition with excess pollens [3,18,23,33]. Supporting this, empirical evidence shows that maternal plants of wind- or self-dispersed species selectively matured seedier fruits ([3,7,18,23,24,34] but see [19]). By contrast, fruits with more seeds may not necessarily be of higher quality for bird-dispersed species because birds do not prefer seedy fruits [35,36]. Therefore, we hypothesize that unlike the cases of self- or wind-dispersed species, selective maturation does not influence seed number per fruit for bird-dispersed species. In general, birds prefer fruits that contain a larger portion of edible parts, such as pericarps and arils, compared with its total seeds [35,36]. Therefore, bird-dispersed plant species would selectively mature larger, but not seedier, fruits. On the other hand, some frugivorous birds are known to be limited in the size of fruits they can ingest [37], and several studies suggest that gape width limits the ability of frugivores to process large fruits [38]. Therefore, larger fruits may not always be preferred by bird dispersers. The results of these previous studies indicate that the definition of “high quality” fruits could differ among species with different dispersal types. Therefore, the effects of selective maturation on fruit traits should also be analyzed by taking dispersal mechanism into consideration.

Compared with numerous studies on self- or wind-dispersed species, only a few studies have investigated the selective maturation of bird-dispersed fruits. Guitián [20,21] investigated the selective fruit abortion process for mahaleb cherry (*Prunus mahaleb*), whose fruits are dispersed by birds and other animals [39,40]. Guitián [20] has experimentally shown that this species selectively matured larger fruits. Additionally, although the number of seeds per fruit was not reported in the article [20], the number of seeds per fruit should have been fixed to one, because *P. mahaleb* fruits are one-seeded drupes [39]. The larger but not seedier fruits may be of higher quality for this bird-dispersed species, and Guitián’s results support our hypothesis that the selection criteria differ among species with different dispersal mechanisms. In another study, Thompson and Dommée [22] investigated jasmine (*Chrysojasminum fruticans* (syn. *Jasminum fruticans*)). The fruits of this species are berries [41], but we are unaware of any reliable scientific literature on the fruit dispersal mechanism of this species. Additionally, Thompson and Dommée did not investigate the fruit traits. Similarity, Collevatti et al. [14] found that pequi (piqui) trees (*Caryocar brasiliense*) selectively abort self-pollinated seeds. Although fruits of this species are drupes [42], the effects of selective abortion on fruit traits was not investigated in their study. Selective abortion was also reported for acorns of oaks (*Quercus ilex* [5] and *Q. serrata* [10]), which are dispersed by birds and animals by means of scatter hoarding after primary gravitational fall [43,44,45,46]. However, because acorns themselves are foods for their dispersers, their fruit traits cannot be directly compared with those of fruits that are primarily dispersed by birds, which is the main focus of the present study. Winsor [47] found evidence of selective fruit maturation for the common zucchini squash (*Cucurbita pepo*, Black Beauty Bush variety), but since the plant in question was a cultivar, and its dispersal agent may be absent among present-day wild taxa [48], their results cannot be directly compared with those of the other cases. Therefore, evidence to support our hypothesis is currently limited and further evidence is needed for bird-dispersed species. In this study, we investigated selective maturation on the fruit traits of elderberry (*Sambucus racemosa* L.) using experimental hand-thinning of inflorescences. We expected results similar to those for another bird-dispersed species *P. mahaleb* [20], i.e., the selective maturation of fruits that are larger but not seedier.

## 2. Results

**Figure 1:**
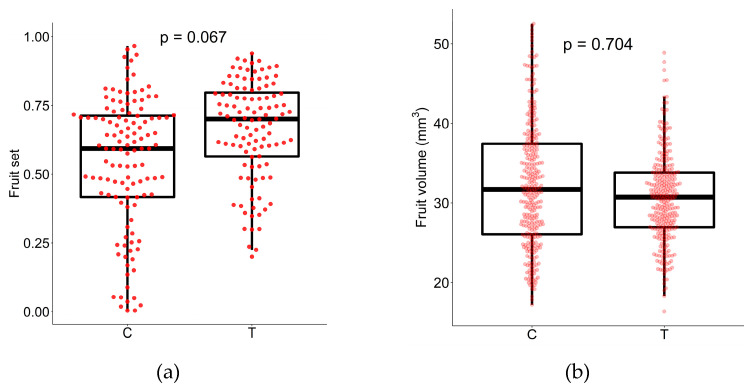
(**a**) Fruit set for inflorescences from the control (C) and treatment (T) individuals (12 control and 12 treatment trees). Each red closed circle indicates one inflorescence (i.e., bee swarm plots superimposed on box plots), and its vertical position indicates the value of the fruit set of that inflorescence. Each circle was shifted in the horizontal direction so as not to overlap with others (*n* = 120 and 108 infructescences for C and T, respectively). The treatment group showed higher fruit set (GLMM (Gamma), *p* = 0.067), supporting the wider-choice hypothesis. (**b**) The volume of the fruits (three treatment (T) and three control (C) trees). Each red circle indicates one fruit (*n* = 300 fruits for each of T and C). No significant difference was found between the two groups (GLMM (Gamma), *p* = 0.704).

We compared the fruit set and fruit traits between individuals in the control (C) and treatment (T) groups. For the treatment group, half of the infructescences on each tree were experimentally removed just after flowering. No manipulation was performed for the control individuals. Inflorescences from the treatment individuals showed a higher fruit set than those of individuals in the control group, though the significance level was slightly higher than the conventional critical level (GLMM (Gamma): *p* = 0.067, Figure 1a). The number of seeds in healthy fruits ranged from two to four, but most fruits (>96%) were three-seeded for both groups, and the mean seed number within each fruit was very similar (C: 2.97 and T: 2.99 seeds per fruits) and did not significantly differ between the two groups (*p* = 0.745, Table 1). Mean fruit volume of the control group was only slightly higher (C: 32.3 and T: 30.7 mm^3^), and no significant difference was found between the two groups (GLMM (Gamma): *p* = 0.704, Figure 1b).

## 3. Discussion

We conducted a literature survey that covers previous studies on selective maturation. Currently, selective maturation has been investigated for at least 30 species, and 13 of these are Fabaceae species (Table 2). An increased fruit set resulting from the removal of flowers is consistent with the results of a previous experiment on *Lotus corniculatus* [18] and indicates the existence of the wide-choice mechanism: individuals with fewer fruit selection options show a higher fruit set rate than individuals with more fruit selection options. In our study, elderberry did not selectively mature ovules that grew into seedier fruits (Table 1). This result is consistent with a previous study of another bird-dispersed species (mahaleb cherry (*Prunus mahaleb*) [20]), and contradicts the results of three different self-dispersed species (*Asphodelus albus* [24]; *Chamaecrista fasciculata* (syn. *Cassia fasciculata*) [3], and *Lotus corniculatus* [18]) and three different wind-dispersed species (*Asclepias speciosa* [23], *Campsis radicans* [7], and *Cochlospermum vitifolium* [34]), in which parental plants selectively matured seedier fruits, and the authors concluded that seedier fruits are of superior quality. Our results and those of these previous studies support our hypothesis that selective maturation differentially affects the fruit traits of species depending on their dispersal mechanism.

Fruit size has been considered as being under conflicting selection [38]. Birds prefer fruits that contain a larger portion of edible parts, such as pericarps and arils, compared with its total seeds [35,36]. Supporting this, Guitián [20] reported that larger fruits were selectively matured, whereas seed number per fruit remained unchanged, after selective abortion in mahaleb cherry (*P. mahaleb*). However, larger fruits may not always be preferred by some bird dispersers. Some frugivorous birds have a limit to the size of fruits they can ingest [37] with gape width limiting the ability of frugivores to process large fruits [38]. Therefore, the effects of selective maturation on fruit traits may also depend on what species of birds disperse the fruit. We found limited effects of selective maturation on fruit sizes (Figure 1b). Individual seeds produced from selective maturation were often, but not always, larger or heavier than seeds produced after random removal (Table 2). However, we did not investigate individual seed size, which also affects dispersal efficiency, although a larger seed may not necessary be of higher fitness because of complex plant-animal interaction including seed dispersal [49] and post-dispersal predation [50]. Therefore, the effects of selective abortion should be investigated for a diversity of species with different plant-animal interactions. Currently, however, most studies are limited to wind- or self-dispersed species (Table 2), and further studies on broader taxa are needed to generalize our findings.

Self-thinning (i.e., spontaneous thinning by maternal plants [18,30]) or natural fruit abscission is a complex process influenced by hormonal regulation and source-sink interactions [30,51,52], but we did not investigate the potential effect of artificial hand-thinning on those interactions. Additionally, artificial removal of fruits may also trigger induced defense, which can also alter the chemical composition of fruits [53]. However, the current physiological studies on fruit abortion of wild plants are much less scarce than those on commercial cultivars that are used for fruit production. Therefore, further studies are needed to clarify the physiological mechanisms underlying selective abortion in wild plants.

## 4. Materials and Methods

### 4.1. Study Species and Sites

Elderberry (*Sambucus racemosa* L. subsp. *kamtschatica* (E.L. Wolf) Hultén) (Adoxaceae) is a winter-deciduous shrub that grows on semi-open sites, such as forest gaps or forest edges. Flowers of this species are hermaphrodite [79]. Fruits of this species are dispersed birds [80,81] and bears [80], and fruits of this particular subspecies is also dispersed by at least one bird species [82].

We conducted this study in 2019 on two sites in Obihiro City in Hokkaido, which is located in a cool-temperate region in Japan. The first site was in the Forest of Obihiro (Obihironomori), which is a plantation forest with a mixture of planted and regenerated trees (42°53′ N 143°09′ E, altitude: 86 m a.s.l.). The second site was the Field Center of Animal Science and Agriculture at Obihiro University of Agriculture and Veterinary Medicine (45°52′ N 143°10′ E, altitude: 79 m a.s.l.). The two sites were ca. 3 km apart from each other and were within 10 km from the Japan Meteorological Agency Obihiro Weather Station (42°52′ N 143°10′ E, altitude: 76 m a.s.l.). The mean annual temperature and precipitation at the weather station during 1998–2017 were 7.2 °C and 937 mm, respectively [83].

### 4.2. Flower Removal Experiment

In 2019, 24 individual trees (12 trees per site) were studied (Table 3). In the experimental garden, all of the individuals had been naturally dispersed and grown under or adjacent to windbreak trees surrounding fields planted with forage crops and pasture plants. The sample trees in the garden were located either in well-lit open spaces or in half-open spaces partially shaded by adjacent taller trees. In the Forest of Obihiro, we were not able to determine whether the sample trees were naturally grown or planted. All sample trees at the forest site were located in well-lit, open spaces. We performed a flower-removal experiment following [18]. To reduce sampling bias, we labeled 12 pairs (6 pairs at each site). Each pair consisted of two individuals of similar height that were located near each other within the same study site. From each of these pairs, we chose one individual as a treatment individual and the other as a control. In June 2019, when the flowers started wilting, we artificially removed 50% of the inflorescences with a pair of long-reach pruning scissors in the treatment group. If the individual had an odd number of inflorescences, we removed one inflorescence more than half. Inflorescences were removed by labeling two adjacent inflorescences as a single pair and then removing one of these inflorescences. The control plants were labeled with plastic labels, but did not manipulate them in any way.

### 4.3. Fruit Set

We sampled 5–10 infructescences per tree for both treatment and control individuals on 1–2 August 2019, once the fruits had matured (Table 3). Any differences in the number of infructescences per tree were due to the fact that some trees only produced a limited number of infructescences. The samples were stored in a refrigerator immediately after sampling until the measurements described below were performed. Following Sutherland [[58], we counted the number of fruits attached to each infructescence and estimated the number of flowers by counting the scars on which ovules or immature fruits had attached but dropped before harvesting. We estimated the number of flowers as the sum of the number of fruits and the number of scars. The fruit set (i.e., fruit-to-flower ratio [58]) for each inflorescence was calculated with Equation (1):

(1) $$\mathrm{Fruit}\;\mathrm{set}\;=\frac{\mathrm{fruit}}{\mathrm{flower}}=\frac{\mathrm{fruit}}{\mathrm{fruit}+\mathrm{scars}}$$

The fruits were sampled just after they matured, but because we did not use nets to prevent birds from removing the matured fruits, the fraction of fruits removed by birds before sampling is unknown.

### 4.4. Fruit Size and Seed Number per Fruit

Using the same set of infructescence samples for the fruit set measurements described above, we counted the number of seeds per fruit for selected fruits (Table 3). Ten healthy fruits were measured for each infructescence, and when the number of fruits on infructescences was less than ten, we sampled all the fruits on that infructescence. Fruit sizes were measured for 600 fruits from 60 infructescences from six individual trees from the Forest site (Table 3). Length was measured in two directions using digital calipers (resolution: 0.01 mm, CD-15AX, Mitutoyo Corp., Kawasaki, Japan). The first length (*l*) was measured as the vertical distance between the bottom and the top of the fruit. The bottom of a fruit was defined as the place with which a fruit attaches to the infructescence. The second measurement was the width (*w*), which was measured as the maximum horizontal width. We calculated the volume of each fruit (*V* (mm*^3^*)) as Equation (2):

(2) $$V=\frac{4}{3}\pi \left(\frac{l}{2}\right){\left(\frac{w}{2}\right)}^{2}$$

Size measurements were taken within two days of the sampling date. This limited the number of fruits for which size measurements were taken (Table 3), but it allowed us to finish the measurements when the fruits were still fresh. Therefore, fruit sizes were only measured for six individual trees at the forest site. For the rest of the trees, fruit size was not measured, but we subsequently counted the number of mature seeds for each fruit in August 2020 using fruit samples stored in a refrigerator. All of the datasets used in this article are available online as Supplementary Materials.

### 4.5. Statistical Analysis

All the statistical analyses described below were performed using the statistical software R v4.0.3 [84] and its packages (“ggbeeswarm” [85], “ggplot2” [86], and “lme4”[87]). The difference in fruit sets between the treatment and control groups was tested using a logistic regression analysis using the generalized linear mixed model (GLMM) employing binomial error distribution (i.e., the fruits either matured or dropped before maturation (as recognized by the scar)) with logit link function [38,88] using the R function *glmer* (family = binomial (link = ”logit”)) [87] and setting the manipulation condition (either treatment or control) as the fixed effect. By referring to [89], we constructed the model of maximal random effects structure instead of choosing random variables via model selection, and we chose all the random effects justified by the design. In the present case, random effects were nested (i.e., inflorescences (infructescences), individuals (tree), pairs, and sites [either the forest or the university]). All these factors were included as nested random intercepts. The random slopes were the pairs and the sites. Because each individual or inflorescence experienced only a single manipulation condition, it was not possible to determine random slopes for these two variables (i.e., unidentifiable model [89]). As shown in the Results section, the difference was not significant (*p* = 0.0667), and reducing some random effects, such as excluding the site effect, often produced significant results (*p* < 0.05). However, because failure to include maximum random effects would have inflated the Type I error [89], we took a conservative approach. The differences in seed number per fruit between the two groups was tested using a GLMM (family = poisson (link = “log”) with the same random effects as described above. The differences in fruit volume between the treatment and control trees were tested using the GLMM (family = Gamma (link = “log”) with basically the same random effects as described above, with the modification that because the fruit volumes were measured only for the individuals from the forest site, site random effects were not incorporated for the analysis. We used a Gamma error distribution because it is used to describe continuous and positive variables [49,90].

## Figures and Tables

**Table 1:** Seed number per fruit.

Number of Seeds Within Each Fruit	Fruit Counts		Relative Frequency (%)	
	Control	Treatment	Control	Treatment
2	40	10	3.44%	0.93%
3	1116	1066	96.04%	98.70%
4	6	4	0.52%	0.37%
Total	1162	1080	100%	100%
Mean seed number per fruit	2.97	2.99		

**Table 2:** A list of species for which selective maturation was investigated (including negative results). A plus sign (+) indicates that the effect of selective maturation increased the value of that trait. For example, for seed number per fruit, a plus sign indicates that maternal plants selectively matured fruits that contained more seeds than aborted immature fruits or ovaries. A minus sign (−) indicates that the opposite was found. A plus–minus sign (±) indicates that mixed results were observed. A zero sign (0) indicates that no effect of selective maturation was observed on that trait. A blank cell indicates that no information on that topic was found in our literature survey. Sp. (species codes): Aa: *Asphodelus albus* Miller (Liliaceae); Af: *Anagyris foetida* L. (Fabaceae); Am: *Agave mckelveyana* Gentry (Asparagaceae); Ao: *Anchusa officinalis* L. (Boraginaceae); Ap: *Achillea ptarmica* L. (Asteraceae); As: *Asclepias speciosa* Torr. (Apocynaceae); Bu: *Bauhinia ungulata* L. (Fabaceae); Cb: *Caryocar brasiliense* Cambess. (Caryocaraceae); Cf: *Chamaecrista fasciculata* (Michx.) Greene (syn. *Cassia fasciculata*) (Fabaceae); Cp: *Cucurbita pepo* cv. Black Beauty Bush (Cucurbitaceae); Cr: *Campsis radicans* (L.) Seem. ex Bureau (Bignoniaceae); Cv: *Cochlospermum vitifolium* (Willd.) Spreng. (Bixaceae); Jf: *Chrysojasminum fruticans* (L.) Banfi (syn. *Jasminum fruticans*) (Oleaceae); Lc: *Lotus corniculatus* L. (Fabaceae); Mj: *Mirabilis jalapa* L. (Nyctaginaceae); Of: *Oreocarya flava* (syn. *Cryptantha flava*) A. Nelson (Boraginaceae); Pc: *Phaseolus coccineus* L. (Fabaceae). Pg: *Pultenaea gunnii* Benth. (Fabaceae). Pm: *Prunus mahaleb* L. (Rosaceae); Qi: *Quercus ilex* L. (Fagaceae); Qs: *Quercus serrata* (Fagaceae); Rs: *Robinia pseudoacacia* L. (Fabaceae); Sa: *Senegalia ataxacantha* (DC.) Kyal. and Boatwr. (syn. *Acacia ataxacantha*) (Fabaceae); Sp: *Senegalia polyacantha* (Willd.) Seigler and Ebinger (syn. *Acacia polyacantha*) (Fabaceae); Sr: *Sambucus racemosa* L. subsp. *kamtschatica* (E.L. Wolf) Hultén) (Adoxaceae); Ss: *Senegalia senegal* (L.) Britton (L.) Britton. (syn. *Acacia senegal*) (Fabaceae); Ts: *Triumfetta semitriloba* Jacq. (Malvaceae); Ue: *Ulex europaeus* L. (Fabaceae); Ug: *Ulex gallii* (Fabaceae). Vc: *Vachellia caven* (syn. *Acacia caven*) (Molina) Seigler and Ebinger (Fabaceae).

Sp.	Fruit Type	Type of Primary Dispersal ^1^	Hand- Removal Performed?	Wider Choice ^3^	Seed Num. per FRUIT	Fruit Size	Seed Size ^2^	Offspring Vigor	Ref. ^4^
Aa	Capsule	Self ^5^	Flowers and fruits		+				[24]
Af	Legume [54]	Self [54,55]	Flowers	Mixed results	±		±		[56]
Am	Capsule [57]		Flowers	No	0				[58]
Ao	Nutlet [59]	Self [60]	Ovules	No			–		[59]
Ap	Achene [61]	Self [60]	Flowers	No	0 Fixed ^6^ [61]			0	[62]
As	Legume	Wind [63]	No		+			Increased germination rate	[23]
Bu	Legume	Self [27]	Fruits		+		+	Increased germination rate	[27]
Cb	Drupe [42]	Gravity, with possible dispersal by large birds [64]	No						[14]
Cf	Legume [65]	Self [65]	Flowers	+	+				[3]
Cp	Pepo [48]	May not exist in the wild [48]	No		+			Rapid germination, increased seedling biomass	[47]
Cr	Capsule [66]	Wind [7,66]	No		+		+		[7]
Cv	Capsule	Wind [67]	No		+				[34]
Jf	Berry [41]		Fruits					Mixed results	[22]
Lc	Legume	Self	Flowers	Yes	+			Increased growth rate	[18]
Mj	Capsule [68]		No		0 Fixed ^6^				[12]
Of	Nutlet [69]	Wind [69]	Ovules					Increased germination rate, but no change in survival rate	[28]
Pc	Legume	Self [70]	Ovules					Rapid germination, increased growth, survival, and reproductive output	[25]
Pg	Legume [71]	Self (primary)	No		0 Fixed ^6^				[31]
Pm	Drupe	Birds and other animals [40]	Flowers		0 Fixed ^6^	+	+		[20,21]
Qi	Acorn	Gravity (primary), animals (secondary) [43,44]	No		0 Acorn			Change neither in germination nor growth rates, but a difference in resource allocation	[5]
Qs	Acorn	Gravity (primary) [45] animals (secondary) [46]	No		0 Acorn				[10]
Rs	Legume	Wind, water [72]	No				+	Increased seedling size	[73]
Sa	Legume	Wind							[74]
Sp	Legume	Wind							[74]
Sr	Drupe	Birds and other animals	Flowers	Yes	0	0			This study
Ss	Legume	Wind							[74]
Ts	Capsule [75]	Epizoochory [76]			+				[15]
Ue	Legume	Self (primary)	No						[26]
Ug	Legume	Self (primary)	No						[26]
Vc	Legume [77]	Self [77]	Flowers		0	0	0	Increased germination rate and seedling survival rate	[19]

^1^ We focused mainly on primary dispersal mechanisms here. Some seeds with elaiosomes may be dispersed secondarily by ants after primary dispersal. ^2^ Individual seed mass or size. ^3^ Wider choice: “Yes” indicates that the percentage of abortion was higher for plants in the natural condition (i.e., wider choice) than those that experienced hand-thinned experiment. “No” indicates that no difference was found between the treatments. ^4^ References for each row, unless otherwise specified within each cell. ^5^ Self-dispersal (autochory) includes dispersal by ballistic or explosive dispersion and/or gravitational fall [78].^6^ Single-ovuled fruits (including acorns).

**Table 3:** Sample sizes.

	Treatment (Inflorescences 50% Thinned)	Control
Total number of trees investigated	12	12
Median tree height (m)	2.98	2.69
Range of tree height (m)	1.70–3.83	1.75–3.85
Total number of infructescences sampled	108	120
Total number of fruits counted for the calculation of the fruit set	16,998	15,036
Total number of fruit scars on the infructescences counted for the calculation of the fruit set	7544	11,626
Total number of fruits investigated for counting seed number per fruit	1080	1162
Total number of trees for which fruit sizes were measured (only for the Forest site)	3	3
Total number of infructescences used for size measurement	30	30
Total number of fruits used for size measurement	300	300

## Data Availability

The data presented in this study are available in the Supplementary Materials.

## Supplementary Materials

All the dataset used in this article are available online at https://www.mdpi.com/2223-7747/10/2/376/s1, S1: dataset.csv. The data may be used with proper citation of this article without contacting the authors.
